# The impact of COVID-19 pandemic on surgical neuro-oncology: A survey from the Italian society of neurosurgery (SINch)

**DOI:** 10.1016/j.wnsx.2023.100233

**Published:** 2023-06-24

**Authors:** Luca Zanin, Tamara Ius, Pier Paolo Panciani, Felice Esposito, Andrea Gori, Marco Maria Fontanella, Maria Pia Tropeano, Antonino Raco, Filippo Flavio Angileri, Giovanni Sabatino, Alessandro Olivi, Vincenzo Esposito, Federico Pessina, SINch NeuroOncology Study Group, Edoardo Agosti, Salvatore Aiello, Denis Aiudi, Danilo Aleo, Roberto Altieri, Rosina Amoroso, Anna Maria Auricchio, Giuseppe Barbagallo, Andrea Barbanera, Giacomo Beggio, Andrea Bianco, Riccardo Boccaletti, Stefano Borsa, Giuseppe Canova, Paolo Cappabianca, Manuela Caroli, Michele Alessandro Cavallo, Francesco Certo, Marcella Chimenti, Franco Chioffi, Valentina Cioffi, Fabio Cofano, Christian Cossandi, Giancarlo D’Andrea, Raffaele De Falco, Alessandro D'Elia, Giuseppe Maria Della Pepa, Alessandro Della Puppa, Attilio Della Torre, Paolo Ferroli, Diego Garbossa, Antonino Germanò, Alessandra Giaquinta, Franco Guida, Maurizio Iacoangeli, Domenico Gerardo Iacopino, Angelo Lavano, Giuseppe Maimone, Vincenza Maiola, Rosario Mauferi, Alessandro Melatini, Mario Moro, Domenico Murrone, Giovanni Muscas, Piero Andrea Oppido, Fabrizio Pignotti, Domenico Policicchio, Piermassimo Proto, Paolo Quaglietta, Antonino Raco, Giulia Renisi, Luca Ricciardi, Francesco Romeo, Marta Rossetto, Alba Scerrati, Andreas Schwarz, Miran Skrap, Carlo Somma, Teresa Somma, Giannantonio Spena, Stefano Telera, Luigino Tosatto, Maria Pia Tropeano, Francesco Volpin, Lorenzo Volpin, Cesare Zoia

**Affiliations:** a Neurosurgery, Department of Medical and Surgical Specialties, Radiological Sciences and Public Health, University of Brescia, Spedali Civili di Brescia, Brescia, Italy; b Neurosurgery Unit, Department of Neurosciences, Santa Maria Della Misericordia University Hospital, Udine, Italy; c Division of Neurosurgery, AOU Sant’Andrea, Department of NESMOS, Sapienza University, Rome, Italy; d Department of Neurosciences and Reproductive and Odontostomatological Sciences, Division of Neurosurgery, University of Napoli "Federico II", Naples, Italy; e Infectious Diseases Unit, Foundation IRCCS Ca' Granda Ospedale Maggiore Policlinico, Via Francesco Sforza 35, 20122, Milan, Italy; f Neurosurgical Department-Humanitas Clinical and Research Center - IRCCS, Via Manzoni 56, 20089, Rozzano, Mi, Italy; g Humanitas University, Department of Biomedical Sciences, Via Rita Levi Montalcini 4, 20090, Pieve Emanuele, Milan, Italy; h Division of Neurosurgery, BIOMORF Department, University of Messina, Italy; i Institute of Neurosurgery, IRCCS Fondazione Policlinico Universitario Agostino Gemelli, Catholic University, Rome, Italy; j Division of Neurosurgery, Mater Olbia Hospital, Olbia, Italy; k Department of Neurosurgery "Giampaolo Cantore"-IRCSS Neuromed, Pozzilli, Italy; l Department of Human Neurosciences-"Sapienza" University of Rome, Italy

**Keywords:** COVID-19, Neuroncology, SINch, Survey

## Abstract

**Background:**

The COVID-19 pandemic and its impact on hospitals' activity and organization has imposed a vast change in standard neurosurgical oncology practice to accommodate for shifting resources.

**Aims:**

This investigation aims to analyse the nationwide capability in reorganizing the surgical neuro-oncological activity during the COVID-19 pandemic to evaluate whether COVID-19-pandemic influenced the surgical management in these patients.

**Method:**

A web-based dataset model organized by the Italian Neurosurgical Society (SINCh) was sent to all the Italian neurosurgical departments in May 2021, requesting to report the types and numbers of surgical procedures performed in the pre-pandemic period (from March 9th 2019 to March 9th 2020) compared to the pandemic period (from March 10th 2020 to March 10th 2021).

**Results:**

This multicentre investigation included the surgical activity of 35 Italian Neurosurgical Departments in a pre-pandemic year versus a pandemic year. During the COVID period, 699 fewer neuro-oncological patients were operated on than in the pre-COVID period. We noted a slight increase in urgency and a more severe decrease in elective and benign pathology. None of these differences was statistically significant. Surgically treated patients who tested positive for SARS-CoV-2 were 36, of which 11 died. Death was found to be COVID-related only in 2 cases.

**Conclusion:**

The reorganization of the Italian Neurosurgical Departments was able to guarantee a redistribution of the CNS tumors during the inter-pandemic periods, demonstrating that patients even in the pandemic era could be treated without compromising the efficacy and safety of the surgical procedure.

## 1 Introduction

The COVID-19 pandemic has had a distressing impact on the National Health System (NHS), causing significant organizational, processional and management struggles.^1^

Besides the respiratory problems,^2^, SARS-CoV-2 has been responsible for a decrease in the number of patients with other conditions who accessed the emergency departments,^3^^,^^4^, including neurosurgical conditions.

Italy was among the first countries overwhelmed by the COVID-19 pandemic in February 2020, recording an extraordinary mortality rate, mainly related to the high percentage of the elderly population.^5^ Considering the tremendous pressure on the healthcare system for the diagnosis and treatment of COVID-19 patients, the Government declared a national lockdown on March 9, 2020^6^

To limit the spreading of the infection and reduce the pressure on the national health service, the Italian Government imposed a nationwide lockdown of all non-essential services between March 10, 2020, and May 3, 2020. Hospitals were forced to reduce elective activities for the more significant part of the period between March and May 2020, limiting patients' access and dedicating most resources to treating symptomatic COVID-19 patients.^7^

In the subsequent inter-pandemic period, from May to October 2020, following the first wave, the number of COVID-19 patients decreased, lockdown measures relaxed, and elective surgeries and clinic services started again.^8^ Between October and November 2020, when the number of COVID-19 patients again increased, the second wave of the COVID-19 pandemic was recorded. During this period, 30–35% capacity of hospitals was reserved for COVID-19 patients, but elective surgeries and outpatient services were ongoing.^9^

Hospitals quickly changed their organization, designing COVID-wards internal paths for COVID patients and specifically dedicated operating rooms.^10^^,^^11^

This study aimed to weigh the effect of the COVID-19 pandemic and the consequent lockdown6 on the treatment of CNS tumors, performing a multicenter analysis in 35 Italian neurosurgical centres with different geographical and structural characteristics. Similar studies have been published worldwide.^12^, ^13^, ^14^, ^15^, ^16^, ^17^

A secondary objective was to investigate how the Italian neurosurgical centres have reorganized their activities to ensure care for patients who needed it.

## 2 Materials and methods

### 2.1 Study population

Thirty-five Italian neurosurgical centres were involved in this study, listed in Table 1. A shared web-based survey organized by the Italian Society of Neurosurgery (SINch®) was submitted to two neurosurgeons in each of the centres involved.^18^ Respondents were asked to use data from their surgical records. Different queries investigating the COVID period, defined from March 10 2020, to March 10 2021, were analyzed, including.

1 the exact number of neuro-oncological surgeries, emergency or elective.
2 the type of tumour pathology.
3 the specific type of surgical procedure performed.

**Table 1:** List of neurosurgical centers participating in the multicenter study with subdivision in North and Central-South groups.

Hospital	City	Group
U.O. Neurochirurgia Ospedale Regionale "F.Miulli"	Acquaviva delle Fonti	Central-South
U.O.C. Neurochirurgia, Dipartimento di Neuroscienze, A.O.U. Friuli Centrale	Udine	North
U.O. Neurochirurgia, Fondazione IRCCS Policlinico San Matteo	Pavia	North
U.O. Neurochirurgia, A.O.U. "Federico II"	Napoli	Central-South
U.O.C. Neurochirurgia Policlinico Universitario di Germaneto	Catanzaro	Central-South
U.O.C. Neurochirurgia, Istituto Clinico Humanitas	Milano	North
U.O. Neurochirurgia, IRCSS Istituto Nazionale Tumori "Regina Elena"	Roma	Central-South
U.O.C. Neurochirurgia Ospedale "A.Perrino"	Brindisi	Central-South
U.O.C. Neurochirurgia A.O.U. Padova	Padova	North
U.O. Neurochirurgia, A.O.U. Ferrara	Ferrara	North
U.O. Neurochirurgia, A.O.U. Policlinico "Paolo Giaccone"	Palermo	Central-South
U.O Neurochirurgia, A.O.U. Maggiore della Carità	Novara	North
U.O. Neurochirurgia, Ospedale Vito Fazzi	Lecce	Central-South
U.O.C. Neurochirurgia Fondazione IRCCS Cà Granda Ospedale Maggiore Policlinico	Milano	North
U.OC. Neurochirurgia, Ospedale Santa Maria delle Grazie	Pozzuoli	Central-South
S.O.D. Neurochirurgia – A.O.U. Careggi	Firenze	Central-South
U.O.C. Neurochirurgia A.O.U. Sassari	Sassari	Central-South
U.O. Neurochirurgia 2 Fondazione IRCCS Istituto Neurologico C. Besta	Milano	North
U.O. Neurochirurgia, Ospedale Fabrizio Spaziani	Frosinone	Central-South
U.O.C. Neurochirurgia A.O. Cosenza	Cosenza	Central-South
U.O. Neurochirurgia, A.O.U. Sant'Andrea	Roma	Central-South
U.O. Neurochirurgia, ULSS2 Marca Trevigiana Ospedale Ca'Foncello	Treviso	North
U.O. Neurochirurgia, Ospedale Civile Santi Antonio e Biagio e Cesare Arrigo	Alessandria	North
U.O. Neurochirurgia, AULSS 8 - Ospedale San Bortolo	Vicenza	North
U.O. Neurochirurgia Ospedale dell'Angelo	Mestre	North
U.O Neurochirurgia, Ospedali Riuniti di Ancona	Ancona	Central-South
U.O. - Neurochirurgia - Ospedale "M. Bufalini" - AUSL Romagna	Cesena	Central-South
U.O. Neurochirurgia, Ospedale di Bolzano	Bolzano	North
U.O. Neurochirurgia Fondazione Policlinico Universitario Gemelli	Roma	Central-South
U.O. Neurochirurgia, Mater Olbia Hospital	Olbia	Central-South
U.O Neurochirurgia, Istituto Neuromed	Pozzilli-Isernia	Central-South
U.O.C. Neurochirurgia, ASST Spedali Civili di Brescia	Brescia	North
U.O. Neurochirurgia, University Hospital, Catania	Catania	Central-South
U.O. Neurochirurgia, A.O.U. Città della Salute e Della Scienza	Torino	North
U.O.C. Neurochirurgia AOU Policlinico “G. Martino”	Messina	Central-South

Comparing results for each answer with the corresponding months of the years 2019–2020 (from March 9, 2019, to March 9 2020), defined as pre-COVID period and used as the control group. We also considered the exact number of COVID-19-positive patients operated on during the COVID period. We also performed a sub-analysis of the results, dividing the participating centres into two macro-categories: north and centre-south, as shown in Table 1, to highlight disparities between different Italian areas.

### 2.2 Statistical analysis

We used Student-tests and ANOVA for continuous variables, and chi-square or Fisher's exact test was used for categorical variables. Continuous data are reported as the mean ± standard deviation. A *p* < 0.05 was considered statistically significant.

Data were collected by considering the sum of single-institution surgical activity in the 12 months before the **COVID-19 pandemic (from March 9 2019, to March 9 2020)** and the sum of single-institution surgical activity during the first year of the **COVID-19 pandemic (from March 10 2020 to March 10 2021).**

Considering the geographical distribution of the participating centres (15 North, 4 Center and 16 South), we compared the surgical procedures in the north versus those in Center + South to balance the results. All statistical procedures were performed using SAS software, version 9.4 (SAS, Cary, NC, USA) and SPSS statistic software, version 26.0 (IBM®)

## 3 Results

**Fig. 1:**
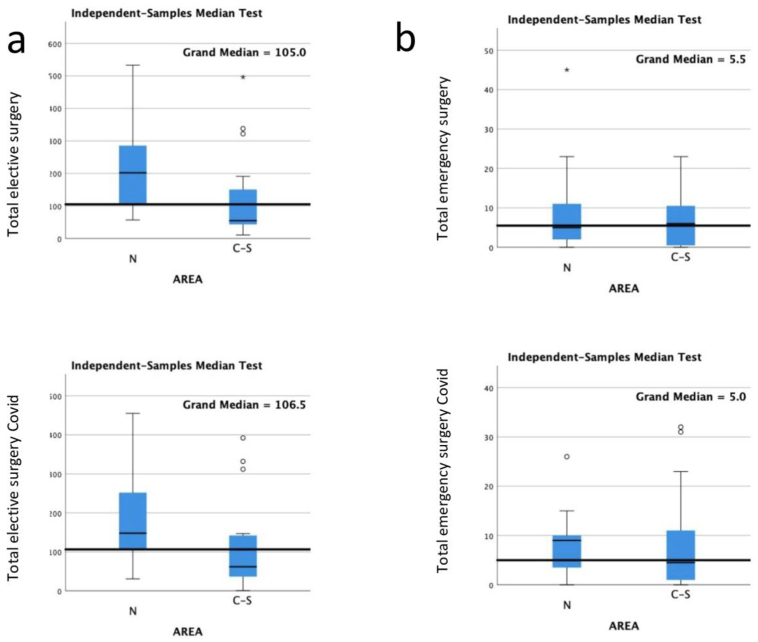
a) shows the difference of means between the total number of elective neuro oncological surgeries in the COVID period and the total number of elective neurooncological surgeries in the pre-COVID period. b) shows the difference of means between the total number of urgent neurooncological surgeries in the COVID period and the total number of urgent neurooncological surgeries in the pre-COVID period. In neither case is statistical significance reached.

**Fig. 2:**
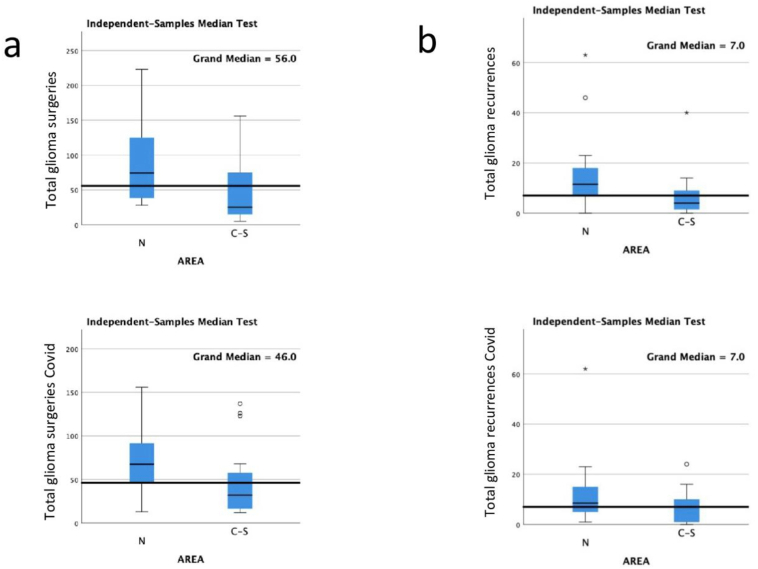
a) shows the difference of means between the total number of first diagnosis gliomas operated during the COVID period and the pre-COVID period. b) shows the difference in means between the total number of glioma recurrences operated during the COVID period and the pre-COVID period.

Thirty-five neurosurgical centres answered the web-based survey released by SINch; 15 were in the country's north, and 20 were in the Center-South. Table 2 shows a direct comparison in the number of patients with CNS tumors undergoing surgery. In the COVID period, we operated 5372 patients, while in the pre-COVID period we operated 6071 patients, with a difference of 699 fewer patients (*p* = 0.5); we carried out a total of 305 emergency neurooncological interventions compared to 244 of the same period in 2019 and 5827 elective surgeries compared to 5067 of the COVID period (*p* = 0.5; *p* = 0.73). Fig. 1 describes a statistical analysis comparing emergency and elective surgeries in the two periods of interest, obtaining no statistically significant results. Table 3 considers the various types of CNS tumors, comparing the COVID period with the pre-COVID period. We observed that first diagnoses of high-grade glioma (HGG) decreased by 135 units, while recurrences decreased by 44. Fig. 2 shows the statistical analysis for first diagnoses and recurrence (*p* = 0.32; *p* = 0.61).

**Table 2:** Types of neuro-oncological surgeries performed in the COVID and pre-COVID period.

Surgery	Patients	%
Emergency neuro-oncological surgeries	244	4%
Emergency neuro-oncological surgeries COVID period	305	6%
Elective neuro-oncological surgeries	5827	96%
Elective neuro-oncological surgeries COVID period	5067	94%
Neuro-oncological pathology operated	6071	
Neuro-oncological pathology operated COVID period	5372

**Table 3:** The table shows the comparison in absolute numbers of different pathologies investigated between the pre-COVID period and the COVID period. LGG: Low-grade glioma; HP: hypothalamic-pituitary lesion.

Pathology	Number	%
HGG first diagnosis	1550	26%
HGG first diagnosis COVID period	1415	27%
HGG recurrence	302	5%
HGG recurrence COVID period	258	5%
LGG first diagnosis	246	4%
LGG first diagnosis COVID period	255	5%
LGG recurrence	81	1%
LGG recurrence COVID period	71	1%
Cranial meningiomas	1447	24%
Cranial meningiomas COVID period	1187	23%
Spinal meningiomas	174	3%
Spinal meningiomas COVID period	123	2%
Meningioma recurrence (cranial e spinal)	118	2%
Meningioma recurrence (cranial e spinal) COVID period	112	2%
Cranial metastasis	607	10%
Cranial metastasis COVID period	644	12%
Spinal metastasis	288	5%
Spinal metastasis COVID period	261	5%
HP lesions	696	12%
HP lesions COVID period	528	10%
HP recurrence	72	1%
HP recurrence COVID period	63	1%
CNS lymphoma	136	2%
CNS lymphoma COVID period	135	3%
Acoustic schwannoma	218	4%
Acoustic schwannoma COVID period	178	3%
Total pre-COVID	5935	
Total COVID period	5230

**Fig. 3:**
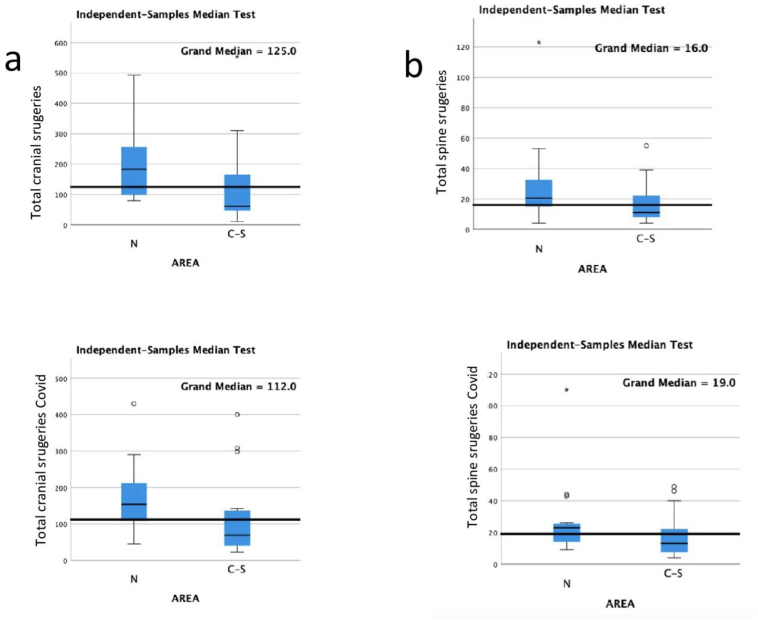
a) shows the difference of means between the total number of cranial surgeries performed during the COVID period and the pre-COVID period. b) shows the same analysis on spinal surgeries.

On the other hand, the diagnoses of low-grade glioma (LGG) were slightly increased by nine units (255 vs 246, *p* = 0.23). The number of *ex novo* diagnoses of cranial meningiomas in the COVID period appears to be significantly reduced compared to the pre-COVID period, with a decrease of 260 patients (1187 vs 1447, *p* = 0.11). In contrast, the number of meningioma recurrences remained substantially stable (112 vs 118, *p* = 0.51). Other pathologies that showed a significant decline in the COVID period were hypothalamic-pituitary tumors, with 168 fewer units (696 vs 528, *p* = 0.11) and acoustic schwannoma, with 40 fewer units (218 vs 178, *p* = 0.10). Instead, the number of CNS lymphomas between the two periods was substantially unchanged. Table 4 examines some specific surgical procedures, among which a significant decline can be noted for awake surgery and transsphenoidal surgery, with a decrease of 99 (225 vs 126, *p* = 0.17) and 171 units (669 vs 498, *p* = 0.12), respectively. Fig. 3 shows our analysis of cranial and spinal surgeries in the COVID and pre-COVID periods, finding no statistically significant differences in either comparison (*p* = 0.42; *p* = 0.95).

**Table 4:** The table shows some types of surgeries in relation to their total number performed in the covid and pre-covid periodsCNS: Central Nervous System; ETT: Endoscopic Transnasal Transsphenoidal.

Procedures	Patients	%
CNS biopsy	380	6%
CNS biopsy COVID period	354	7%
Awake surgery	225	4%
Awake surgery COVID period	126	2%
ETT surgery first diagnosis	669	11%
ETT surgery first diagnosis COVID period	498	9%
ETT surgery recurrence	90	1%
ETT surgery recurrence COVID period	70	1%
Total pre-COVID procedures	6071	
Total COVID procedures	5372

**Fig. 4:**
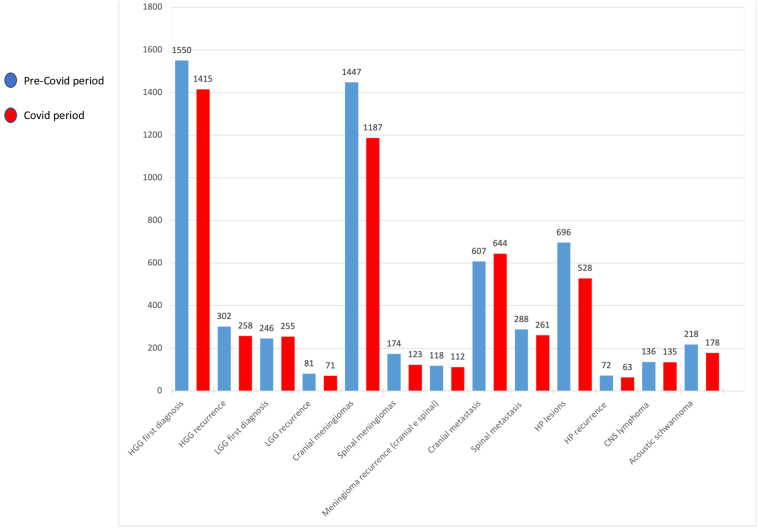
shows the trend of various neurooncological pathologies in Italy during the pre-COVID period (blue bar) and the COVID period (red bar).

**Fig. 5:**
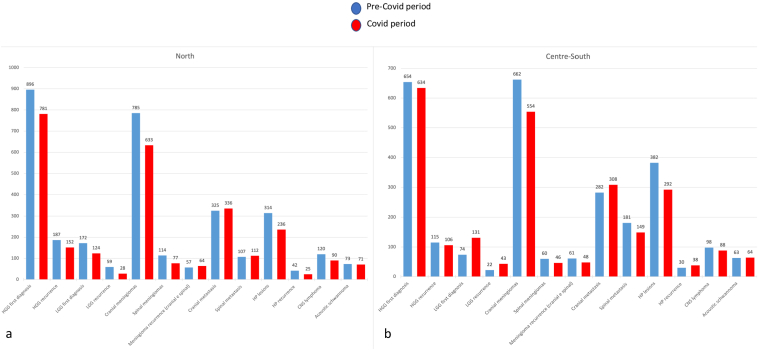
a) and b) show the comparison between two histograms reporting the trend of different neurooncological pathologies during the pre-COVID period (blue bar) and the COVID period (red bar) in North e Central-South of Italy.

**Fig. 6:**
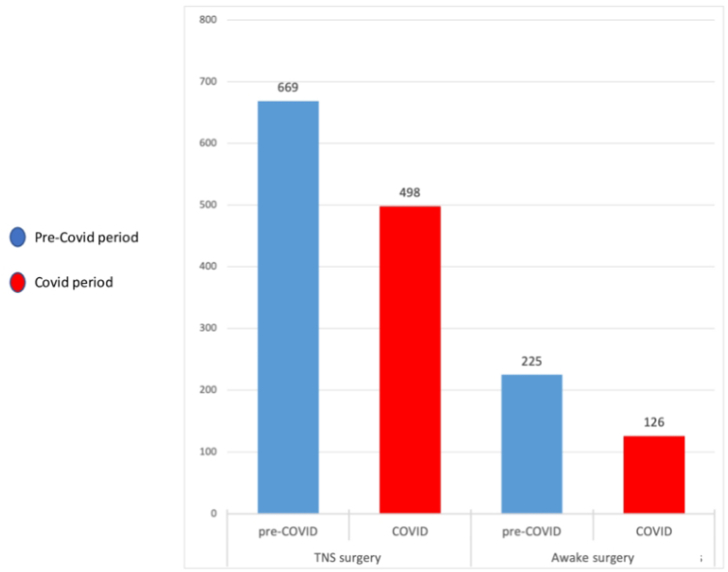
Number of TNS and awake surgery procedures during the pre-COVID (blue bar) and COVID period (red bar).

The analysis between the Northern group and the Central-Southern group essentially showed a more substantial number of interventions carried out in the North group, with the differences reduced in the number of emergency surgeries, both in the pre-COVID and in the COVID period, as shown in Fig. 4, Fig. 5. The reduction in medians during the COVID period also appears to be mirrored in the two groups, except for the total cranial surgeries, which appear to decrease more in the northern group. Fig. 6 explicitly shows the trend of the TNS and awake procedures during the two periods under comparison. The total number of positive patients operated in the COVID period was 36, of which 11 died. The postoperative death was found to be COVID-related only in 2 cases.

## 4 Discussion

From the beginning of the COVID-19 pandemic, several hospitals had to reorganize activities, managing the growing number of patients with respiratory disease, closing routine outpatient visits and postponing elective surgery.^19^^,^^20^ In some hospitals, surgeons also had to serve in COVID wards. This situation led to the definition of "collateral damage" due to COVID-19, with several patients no longer having access to the care they would typically need. This condition occurred in several medical fields.^3^^,^^4^^,^^21^ However, from a comprehensive analysis of the data collected from our web-shared survey, we can observe how the situation of the Italian neurosurgical reality was less complicated than it initially appeared. From the first days of the pandemic, the health system in various Italian regions organized a "hub & spoke" method,^22^, to manage and adequately move patients, guaranteeing the necessary assistance as much as possible. The great demand for anesthesiologists and intensive care operators due to the management of respiratory pathology led to the closure of most elective operating rooms. Despite this, the emergencies continued to be carried out regularly, and it is evident how the total number of emergencies during the COVID period increased compared to the previous year, although not reaching statistical significance. Our data show that in almost all centres, for nearly all neuro-oncological pathologies, there has been a reduction in the number of patients operated on, with rare exceptions. However, these reductions were not statistically significant in our analysis. To explain our results, we can assume that the COVID period in Italy is divisible into four phases: 1) first wave (from March 9 2020, to May 18 2020), when the pandemic hit the NHS hardest, leading to a national lockdown, 2) inter-pandemic period (from May 19 to October 7 2020) following the first wave, when the number of COVID-19 patients decreased, lockdown measures relaxed, elective surgeries and clinic services started again, 3) second wave (from October 8 to December 31 2021) when the number of COVID-19 patients again increased. During this period, our NHS was able to reserve 30–35% capacity of hospitals for COVID-19 patients and still keep ongoing elective surgeries and outpatient services,^9^, 4) post-pandemic period (from January 1 2021, to March 10 2021), where the pandemic has stabilized, until it subsides with the arrival of spring at the end of March 2021. Our NHS, during the interpandemic periods, has managed to redistribute the neuro-oncological patients who had not been treated during the pandemic's acute phases, allowing only a slight reduction in the number of interventions performed compared to the previous year.

Furthermore, the sub-analysis we conducted between the North and Central-South macro-regions showed a numerical difference in surgical interventions already present before the pandemic. This difference can be explained by the fact that several centres of the country's most populated regions (such as Lombardia, Piemonte and Veneto) participated in the proposed survey. It is interesting to note how the numbers dropped symmetrically in the north and the center-south during the COVID period. However, the northern regions, especially Lombardia, were hit the hardest in the early stages of the pandemic.

We observed how the most significant decrease occurred in benign and elective pathology, while for urgent pathology, there was a slight increase in cases (e,g. cranial meningiomas: 1447 in the pre-COVID period vs 1187 in the COVID period, with a drop of 260 units). In neither of the two comparisons, a statistically significant result was obtained. However, the analysis shows how the period of severe stress of the NHS led to a preference for acute disease over elective one and malignant disease over benign one.

A similar argument applies to CNS biopsies, which often require subsequent chemotherapy, perhaps not easily obtainable during the COVID period's maximum crisis. For this reason, they were postponed to moments of more accessible care for the patient.

Instead, a different situation was seen for cranial metastases, which appear to be slightly increased during the COVID period. This is perhaps because they often present in an acute symptomatic way, with seizures or intracerebral bleeding, leading the patient to the emergency room and treated as emergencies.

In addition, it is essential to note that the COVID-19 pandemic caused a significant decrease in surgical activity, especially during the first wave. This forced a subsequent reorganization with a rescheduled procedure, which implied a delay in other neurosurgical pathologies. This collateral damage of the COVID-19 pandemic is probably underestimated and difficult to be traced.

During the COVID-19 period, there was an essential decrease in TNS procedures and Awake surgery, as expected.

### 4.1 Awake surgery

In the COVID-19 pandemic year, a decrease of 2% was recorded for awake craniotomy. This data summarizes the effect of multiple reasons. There has been a growing debate about carrying out awake craniotomy surgeries during the COVID 19-pandemic, not only due to airway management but also to the close patients' proximity to the team in the operating theatre.

Awake surgery carries indeed a theoretically high risk of viral transmission, further worsened by the presence of many people in the operating theatre.

Awake craniotomy carries additional concerns, including the safety of the patient close to multiple staff members, the possibility of transmission of COVID-19 to staff where a pre-operative swab has been falsely negative, and the presence of additional staff/equipment in the theatre to perform necessary testing.

Furthermore, awake surgeries for relatively non-urgent pathologies (e.g. Low, grade gliomas) had been deferred as per SBNS/BNOS guidelines^23^^,^^24^

### 4.2 Endoscopic transnasal transsphenoidal (ETT)

In the COVID-19 pandemic year, a decrease of about 2% was recorded for ETT surgical procedures.

Scientific societies identified the transnasal skull base surgery, transoral and transfacial corridors, as the riskiest for the diffusion of COVID-19^25^^,^^26^ and recommended sparing the opening of paranasal cavities and mastoids during transcranial corridors. Different protocols were recommended to reduce the pooling of secretions by minimizing irrigation, utilizing an evacuation suction, and considering the placement of a throat pack.^27^ Some authors have also shown that electrocautery and ultrasonic devices, such as Sonopet (Stryker), led to aerosolizing viral particles. A suction evacuation was suggested if electrocautery is necessary for hemostasis.^27^ Given the high risk of spreading the virus with this type of surgery, many precautions have been used as scalpels affixed to long handles to avoid aerosolization associated with electrocautery and favour nonabsorbable packing, which is removed without endoscopy.^27^ Considering all the endorsed restrictions looks notable that the decrease was only 2%.

### 4.3 Study limitations

Our study was drawn from a web-based survey that did not consider the chronology of the different phases of the COVID period, so it is not possible to know precisely how the numbers varied between the first and second waves and the interpandemic periods. It is challenging to quantify the collateral damage of the COVID-19 pandemic as factors that are not easily quantifiable emerge, such as the patient's fear of going to the Emergency Department because of the contagion and a possible tendency to underestimate symptoms that in a standard period would have found a quicker outpatient response. More studies are needed in the coming years to clarify this issue. One of our study's main limitations is the lack of a shared national reorganization protocol, especially for the first wave. However, it is interesting to note that there are no significant differences between regions which were more affected than others in terms of numbers. Our data, based on an online survey, do not report the outcome of surgeries. Therefore, it is impossible to establish whether mortality from the neurosurgical disease increased during COVID.

Another potential limitation is the lack of participation of all the Italian neurosurgical centres. Although 35 responding centers represented almost 30% of the neurosurgical centers active in Italy, the remaining centers may have different perspectives and experiences that were not captured in the survey.

## 5 Conclusion

The Italian Neurosurgical Departments were reorganized during the COVID-19 pandemic, which allowed for the nationwide treatment of CNS tumors without compromising surgical efficacy and safety. The impact of the pandemic on Surgical Neuro-Oncology in Italy was serious, but the system was not overwhelmed. While there was a slight decrease in some departments, it was justified by the geographic redistribution of pathology management and the reduction of available resources. The decrease in the number of operated patients was not statistically significant, but further studies are needed to investigate whether it corresponds to an increase in mortality.

## CRediT authorship contribution statement

**Luca Zanin:** Conceptualization, Data curation, Formal analysis, Writing – original draft, Writing – review & editing. **Tamara Ius:** Conceptualization, Data curation, Formal analysis, Supervision, Validation, Writing – review & editing. **Pier Paolo Panciani:** Conceptualization, Data curation, Investigation, Methodology, Supervision, Validation, Visualization, Writing – original draft, Writing – review & editing. **Felice Esposito:** Data curation, Formal analysis, Methodology, Software, Validation. **Andrea Gori:** Conceptualization, Data curation, Formal analysis, Methodology, Supervision, Validation. **Marco Maria Fontanella:** Supervision, Validation, Visualization. **Maria Pia Tropeano:** Data curation, Investigation, Methodology, Validation. **Antonino Raco:** Data curation, Supervision, Validation. **Filippo Flavio Angileri:** Conceptualization, Data curation, Investigation, Methodology, Supervision, Validation, Visualization, Writing – review & editing. **Giovanni Sabatino:** Conceptualization, Investigation, Methodology, Supervision, Validation, Writing – review & editing. **Alessandro Olivi:** Conceptualization, Data curation, Investigation, Methodology, Supervision, Validation, Visualization. **Vincenzo Esposito:** Supervision, Validation, Writing – review & editing. **Federico Pessina:** Conceptualization, Data curation, Methodology, Validation, Visualization, Writing – original draft, Writing – review & editing. **Edoardo Agosti:** Data curation. **Salvatore Aiello:** Data curation. **Denis Aiudi:** Data curation. **Danilo Aleo:** Data curation. **Roberto Altieri:** Data curation. **Rosina Amoroso:** Data curation. **Anna Maria Auricchio:** Data curation. **Giuseppe Barbagallo:** Data curation. **Andrea Barbanera:** Data curation. **Giacomo Beggio:** Data curation. **Andrea Bianco:** Data curation. **Riccardo Boccaletti:** Data curation. **Stefano Borsa:** Data curation. **Giuseppe Canova:** Data curation. **Paolo Cappabianca:** Data curation. **Manuela Caroli:** Data curation. **Michele Alessandro Cavallo:** Data curation. **Francesco Certo:** Data curation. **Marcella Chimenti:** Data curation. **Franco Chioffi:** Data curation. **Valentina Cioffi:** Data curation. **Fabio Cofano:** Data curation. **Christian Cossandi:** Data curation. **Giancarlo D’Andrea:** Data curation. **Raffaele De Falco:** Data curation. **Alessandro D'Elia:** Data curation. **Giuseppe Maria Della Pepa:** Data curation. **Alessandro Della Puppa:** Data curation. **Attilio Della Torre:** Data curation. **Paolo Ferroli:** Data curation. **Diego Garbossa:** Data curation. **Antonino Germanò:** Data curation. **Alessandra Giaquinta:** Data curation. **Franco Guida:** Data curation. **Maurizio Iacoangeli:** Data curation. **Domenico Gerardo Iacopino:** Data curation. **Angelo Lavano:** Data curation. **Giuseppe Maimone:** Data curation. **Vincenza Maiola:** Data curation. **Rosario Mauferi:** Data curation. **Alessandro Melatini:** Data curation. **Mario Moro:** Data curation. **Domenico Murrone:** Data curation. **Giovanni Muscas:** Data curation. **Piero Andrea Oppido:** Data curation. **Fabrizio Pignotti:** Data curation. **Domenico Policicchio:** Data curation. **Piermassimo Proto:** Data curation. **Paolo Quaglietta:** Data curation. **Antonino Raco:** Data curation. **Giulia Renisi:** Data curation. **Luca Ricciardi:** Data curation. **Francesco Romeo:** Data curation. **Marta Rossetto:** Data curation. **Alba Scerrati:** Data curation. **Andreas Schwarz:** Data curation. **Miran Skrap:** Data curation. **Carlo Somma:** Data curation. **Teresa Somma:** Data curation. **Giannantonio Spena:** Data curation. **Stefano Telera:** Data curation. **Luigino Tosatto:** Data curation. **Maria Pia Tropeano:** Data curation. **Francesco Volpin:** Data curation. **Lorenzo Volpin:** Data curation. **Cesare Zoia:** Data curation.

## Declaration of competing interest

The authors declare that they have no known competing financial interests or personal relationships that could have appeared to influence the work reported in this paper.
